# Aegrescit medendo: orthopedic disability in electrophysiology - call for fluoroscopy elimination—review and commentary

**DOI:** 10.1007/s10840-022-01173-5

**Published:** 2022-03-08

**Authors:** Donald S. Rubenstein, Benjamin B. Holmes, Joseph A. Manfredi, Matthew S. McKillop, Peter C. Netzler, Chad C. Ward

**Affiliations:** 1 Carolina Cardiology Consultants–EP Division, Prisma Health, 701 Grove Rd., Greenville, SC 29605 USA; 2 Baptist Medical Center Jacksonville, 800 Prudential Dr., Jacksonville, FL 32207 USA

## Introduction

Aegrescit medendo, the remedy is worse than a disease, was first described in book XII of the Aeneid [1]. Fluoroscopy has been a necessary evil for the interventional electrophysiologist. The use of lead aprons to mitigate rare fatal cancers has created an epidemic of orthopedic disability. The rapid ascent and technological progress in the field of electrophysiology have resulted in increased diagnostic precision, improved procedural success rates, and improved patient survival. Electrophysiology (EP) researchers and industry must align in their efforts to harness that innovation and prioritize the health of ourselves and our staff, while maintaining safe and effective patient procedures. We provide a review of interventional cardiology radiation/fluoroscopy exposure and then a step-wise approach to completely eliminate fluoroscopy during electrophysiologic ablation (EPA) procedures and the implantation of new cardiac rhythm management (CRM) devices.

Fluoroscopy is a continuous live x-ray imaging technique utilizing ionizing radiation that passes through the patient to visualize internal body structures. Following the first transvenous method to implant pacing devices in 1963, fluoroscopy had been the primary cardiac imaging tool to complete these procedures [2]. Two categories of risk reduction are described for the emitted radiation during fluoroscopy. These include methods to decrease either detrimental stochastic effects (DSE, future cancers) or detrimental deterministic effects (DDE, immediate dose-dependent cellular damage) to the patient or lab personnel [3]. One of these risk-mitigating strategies is that all laboratory personnel must wear heavy lead aprons.

**Fig. 1:**
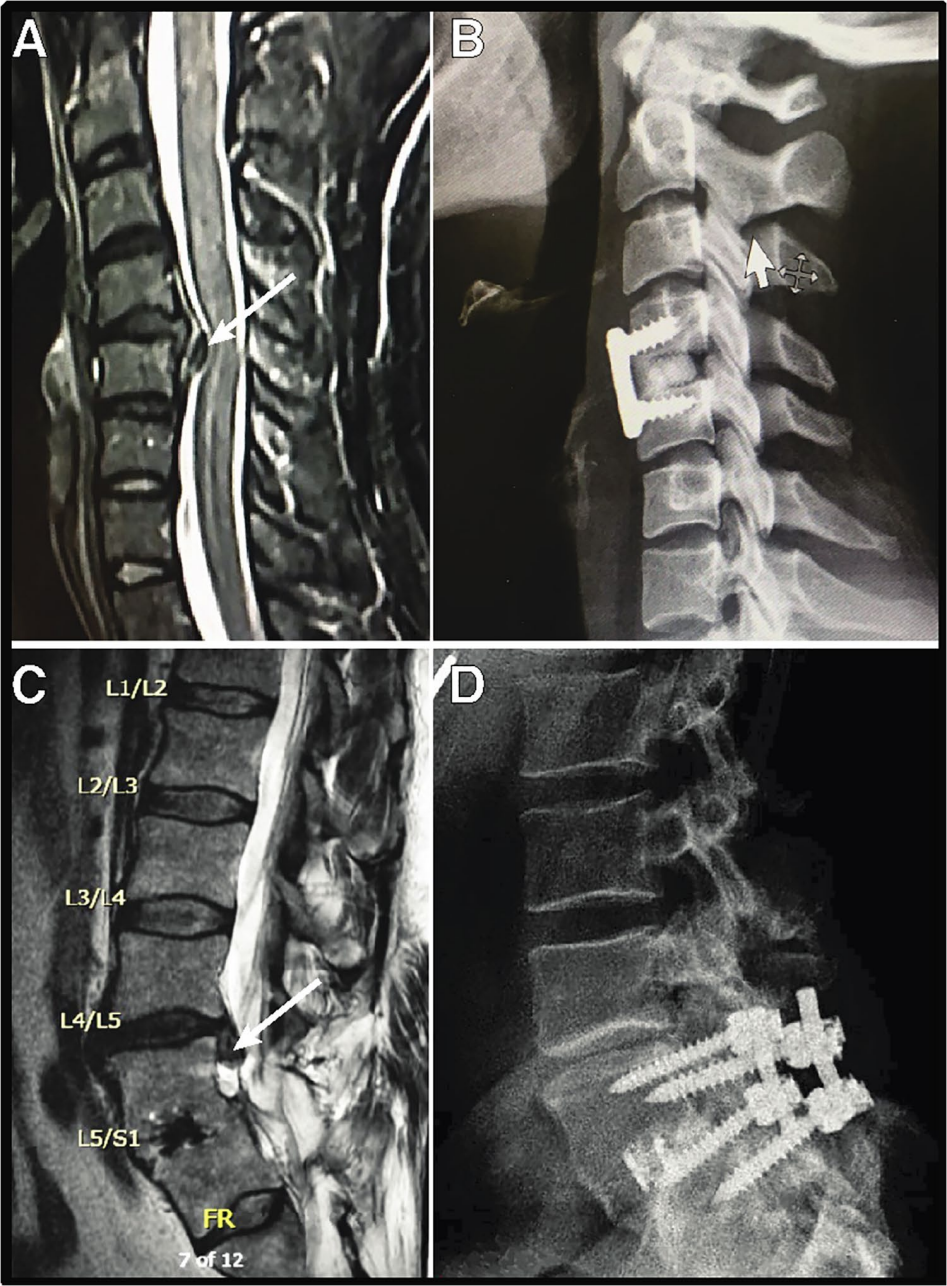
**A** MRI of the cervical spine of CRM device representative only a few years after starting employment. Arrow points to the cervical spine disc herniation. **B** Lateral spine x-ray following repair of herniation in **A**. **C** MRI of the lumbar spine of electrophysiologist with new disc herniation at L4-L5. A prior repair is seen with fusion and titanium cage at L5-S1 that was required approximately 5 years after starting practice. **D** Sagittal view of the lumbar spine of **C**

The consequential orthopedic injury risk from the continued use of lead garments is brought to light. We categorize this risk as detrimental orthopedic effects (DOE). The donning of lead aprons during these daily and long procedures has resulted in the rapidly progressing prevalence of severe musculoskeletal disorders among electrophysiologists. Because DOE has a much greater prevalence and hazard to EP physicians and staff (Fig. 1), DOE is prioritized and appropriately placed alongside DSE and DDE. Major advances in arrhythmia mapping technology by both electroanatomic mapping (EAM) and intracardiac echo (ICE) have provided the ability to eliminate fluoroscopy completely in all forms of cardiac ablation [4–8]. We call upon the EP communities, societies, training programs, and industry to reach freedom from dependency upon fluoroscopy by 2030. The ultimate aim is to eliminate fluoroscopic ionizing radiation use during ablation and implant procedures, eliminate all radiation risks to patients and staff, and thereby eliminate secondary occupational DOE risks of wearing the protective heavy lead aprons each day.

## The rise and fall of EP fluoroscopy

### The benefits

To reduce procedural risks while treating cardiac arrhythmias, imaging methods have gradually evolved from direct surgical visualization to virtual reconstruction of 3D cardiac chambers and their conduction pathways. In 1968, the first open-heart surgery provided direct vision to sever an accessory pathway of a patient with Wolff-Parkinson-White syndrome [9]. Severe congestive heart failure was a common comorbidity in patients suffering from medicine refractory tachycardias, thus making them a too high risk to undergo surgical treatment. This gave rise to closed-chest procedures that, in 1982, proved that wire catheters could deliver high-energy electrical shocks that could provide the similar desired permanent disruption of a rapidly conducting atrioventricular node [10, 11]. Fluoroscopic visualization of the placement of these temporary catheters alongside less traumatic radiofrequency energy quickly propelled the EP field to confront almost all forms of cardiac arrhythmias. Concomitantly, rapid advances were being made in pacemakers and defibrillators, both of which required fluoroscopic imaging for the precise placement of their leads. Within 10 years, concerns surfaced on the effects of accumulating exposure to harmful radiation in both patients and physicians [12]. General guidance was provided to limit fluoroscopic times to less than an hour and limit total procedure time to less than 5 h [12, 13]. Fluoroscopy use in the early years of EP procedures was accomplished by either being allowed shared time in a cardiac catheterization lab, often at the end of the day, or by having access to a procedure room with a portable C-arm. The construction of specialized EP labs evolved from single-plane fluoroscopy to biplane, and even rotational arms. By 2014, the demand for hospitals to construct new and complex EP labs led to a formalized consensus statement with a focus on safety, especially from fluoroscopy [14].

### The risks—detrimental effects

EP procedural and fluoroscopic times are a function of the complexity of the arrhythmia, the chamber location, the number of temporary catheters or permanent leads to be placed, the accuracy of the mapping system, the number of ablation lesions or lesion sets, and the method of ablation. Historically, the elimination of an accessory pathway with just a few discrete lesions could be completed within a relatively short total procedure time. Prior to the development of alternate imaging, fluoroscopy-guided elimination of an accessory pathway averaged 44 min [12]. More complex arrhythmias, such as atrial fibrillation and ventricular tachycardia, that required more extensive and precise mapping with multiple lesion sets often resulted in procedure durations of several hours. Similar to ablation, the procedure times and duration of ionizing radiation exposure increased with the complexity of CRM devices. Radiation exposure increased from single lead to dual lead, with the highest exposure recorded with cardiac resynchronization (CRT) devices [15–17]. The radiation exposure to patients during a CRT implant resulted in a 2–9 times greater exposure than any other device implant [17–20]. Recent data from the RADAR study showed that the DDE radiation effects from atrial fibrillation ablation were comparable to implant procedures of CRT devices [21]. Increased DNA damage was identified in circulating lymphocytes and monocytes as measured by the standardized comet assay. Radiation damage to these cells was seen from either an ablation procedure for atrial fibrillation or from CRT device implantation. The damage to DNA in these cell lines took 3 months to recover. In one study, due to accumulating radiation exposure to the implanting physician’s right hand, the authors not only recommended CRT implantation to be limited to a yearly number, but also recommended avoiding implanting devices on the patient’s right side [18]. It was estimated that the DSE from performing ablation procedures at a frequency of about 360 cases a year would result in an added lifetime risk of a lethal cancer for 1 in 92 EP physicians [22]. Applicable risk data from interventional cardiologists have shown that these physicians are at a significantly higher risk to develop radiation-induced cataracts and brain and neck tumors [23, 24].

### Metrics—procedural radiation reduction and the missing lead “apron-time”

Medical personnel, physicians, and staff present in procedures requiring fluoroscopic imaging are among the occupations with the highest radiation exposure [25]. Lead aprons are worn to mitigate this risk. Strict federal and institutional guidelines have been established to limit occupational radiation exposure. Exposure is closely measured with dosimetry badges required to be worn by all physicians and staff [26, 27]. Extensive safety data is collected with each procedure, including fluoroscopic equipment use, ionizing radiation emission, procedural times, and radiation dose. Many EP labs have upgraded their x-ray equipment to program lower frame or pulse rates. In the most aggressive attempt to overhaul all methods to minimize patient and physician exposure, an ultralow-dose radiation protocol was adopted in one German hospital for all pacemaker and defibrillator implants [28]. Through the combined use of reduced pulse width and rate of emission, increased thickness of copper filters, reduced detector entrance dose, and an optimization of postprocessing image settings, the physicians were able to reduce the effective dose exposure by 59%.

As technology advanced, finally giving rise to treatments for even the most complicated arrhythmia patients, procedure times often lengthened. Very little progress has been made for personal protective garments to shield the physician and staff. Lead aprons are worn to protect the cumulative irradiation risks of all-cause cancer and mortality that have been documented with cardiovascular and electrophysiologic interventionalists. Lead aprons with thicknesses 0.25 to 0.5 mm weigh about 12 to 25 pounds, respectively. The thicker, heavier lead aprons provided far greater prevention of radiation transmission [29]. The aprons are worn under the sterile gowns during the entire procedure. Durations to implant CRT device commonly extend from 1.5 to 2.5 h for CRT device implants [15, 30], while ablation of atrial fibrillation or ventricular tachycardia (VT) may range from 2.5 to 6 h [31–33]. Hanging lead aprons from ceiling-mounted devices makes EP lab construction cumbersome while not protecting the EP lab support staff or the industry device representative. Hanging lead shields have taken on creative shapes, hinges, and armholes, designed to decrease the primary operator’s DSE, DDE, and DOE while many—including lab staff and anesthesia personnel—are still subjected to wearing heavy lead aprons.

No metric exists to track the cumulative occupational load-bearing burden of donning heavy lead aprons. Apron-time, or the amount of time that the lead apron is worn during an EP procedure, is primarily an all-or-none time parameter directly correlated to the length of the procedure. To our knowledge, no study within procedural electrophysiology currently or in the past has collected data on lead apron-time, musculoskeletal disease (MSD), or DOE. Despite progress in decreasing radiation exposure, lead apron-time remains unchanged. A recent review analyzed the work-related MSD among at-risk physicians [34]. These studies included interventional radiologists, surgeons (general, orthopedic, and plastic surgeons), and interventional cardiologists (including electrophysiologists). Degenerative spine disease, defined specifically as spondylosis, spondyloarthropathy, herniated or ruptured disc, or radiculopathy, increased in prevalence among interventional cardiologists from 8 in 1997 to 35% in 2015 [35]. The most common site affected was the lumbar and cervical spine (Fig. 1). The prevalence of MSD among interventional cardiologists quadrupled in less than 20 years [36]. The ability to map and treat even more complex arrhythmias has resulted in even more prolonged procedure times. Lengthy procedure times have catapulted the specialty of electrophysiology to be one of the highest risk specialties to experience and suffer from MSD.

Comparing cardiac and EP interventionalists to non-interventionalists, the interventionalists had a 10% higher risk of radiation-related illness while a > 50% higher risk of orthopedic injury [37]. An astounding 49% reported an orthopedic injury involving the spine (cervical and lumbar), hip, knee, and ankle. The prevalence in this Canadian survey of interventional electrophysiologists for lumbar spondylosis was 25.9% and cervical spondylosis at 20.7%, showing a marked higher trend with years of occupation [37]. 1997 was the last year the CDC studied work-related MSDs [38]. At that time, MSD was the highest cause of disability, resulting in it being the number one cause of absenteeism in all healthcare workers. The impact to the nation economically back then was estimated to be an annual loss of $13 to $20 billion. Specific to physicians, work-related MSD resulted in 9% of physicians requiring a leave of absence, practice restriction or modification, or early retirement [37, 39]. Although the lead aprons may protect against 1 in 92 EP physicians from a lethal cancer [22], they cause at least 1 in 3 EP physicians to suffer severe pain from an MSD, and most will likely require at least temporary disability, if not major surgery [37]. These statistics could likely be extrapolated out to EP lab staff. There are 4–5 EP lab support personnel in a typical ablation or implant procedure. These individuals share the cumulative orthopedic risk of occupational lead use leading to absence or early retirement [40, 41]. Utilizing a metric of apron-time would provide new and essential data to elucidate the risks of DOE further.

## The obsolescence of fluoroscopy—transition to alternate imaging tools

### Ablation procedures are first to phase out fluoroscopy

**Fig. 2:**
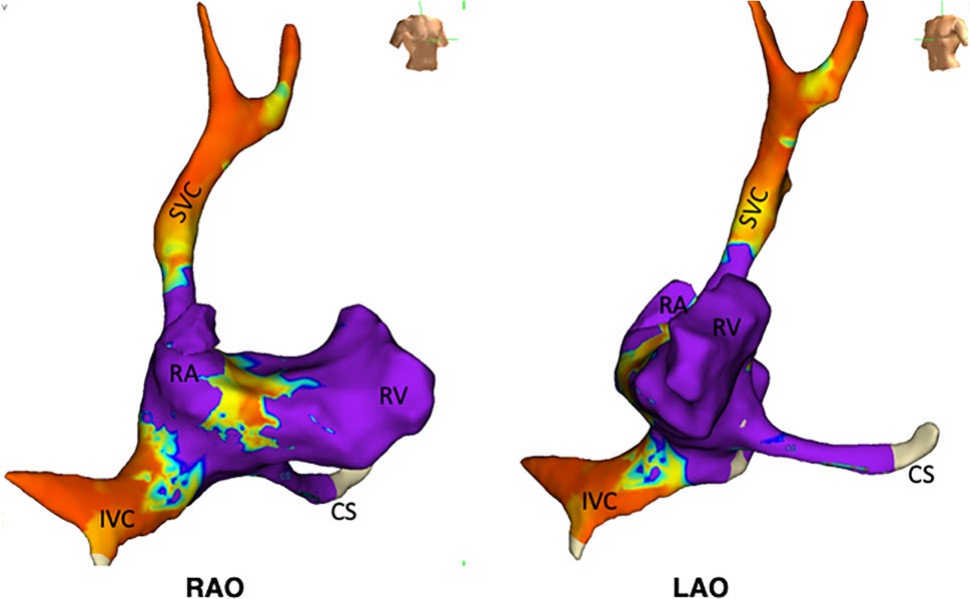
Example of a 3D electroanatomic voltage map of the right atrium and right ventricle RAO and LAO positions constructed with HD Advisor Mapping Catheter. Purple identifies regions of highest amplitude voltages representing good targets for CRM lead positioning. SVC, superior vena cava; IVC, inferior vena cava; RA, right atrium; RV, right ventricle; CS, coronary sinus

**Fig. 3:**
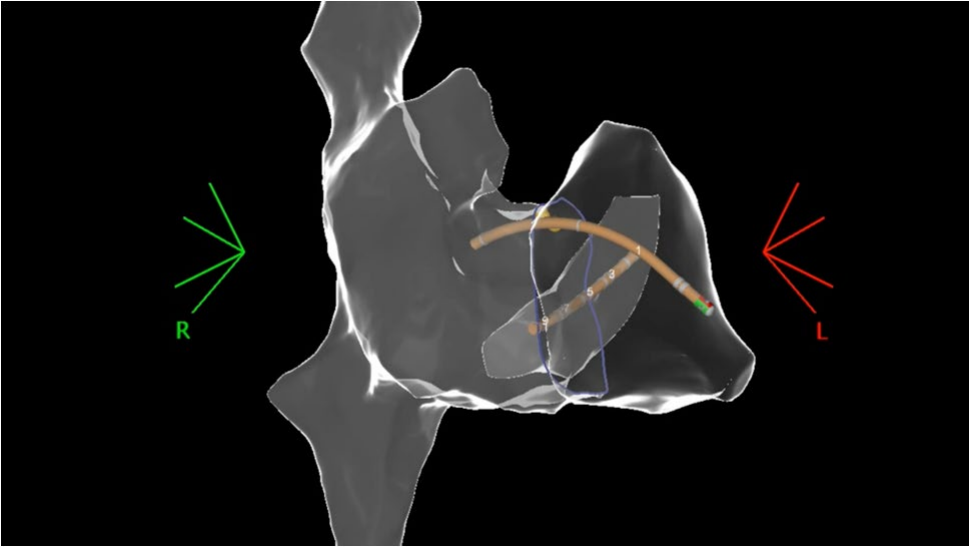
Example of a transparent 3D anatomic map with Carto EAM of right atrium and ventricle with temporary electrophysiologic pacing catheters in the right ventricle and coronary sinus. Double click on the image to animate rotation

During EP procedures, the physician closely observes live fluoroscopic images as electrophysiologic wires are advanced from their vascular access points to specific positions within the cardiac chambers. For arrhythmia ablation procedures, the wires are placed in the cardiac chambers only temporarily, commonly moved throughout the chambers collecting wavefront activation measurements and applying either radiofrequency ablation or cryoablation lesions. Two U.S. Food and Drug Agency (FDA)-approved alternate forms of visualization, EAM and ICE, are well-accepted methods used daily. EAM has been the workhorse for mapping cardiac chambers and ablating most arrhythmias. The two most commonly used commercial EAM systems are EnSite (Abbott Medical, Abbott Park IL) and Carto (Biosense Webster, Inc., Irvine, CA). The acquired 3-dimensional virtual structural image created by EAM is a fixed shell structure with precise site-specific color-coded voltage change recordings (Figs. 2 and 3). If a heart rhythm is stable, following the same path beat-to-beat, then the local time-dependent voltage changes can be processed to allow visualization of the electrical wavefront as it may propagate across the myocardium. Real-time visualization of the catheter positions allows the safe repositioning of the wires within the borders of a fixed virtual image shell. Ultrasonography, and more specifically ICE, on the other hand, provides a live 2-dimensional image slice of cardiac borders of different densities with a probe that is easily deflected and rotated. In real time, one can visualize the cardiac border chamber border limits, interatrial septal motion, valvular motion, and pericardial space.

It was not long after the introduction of EAM that the notion of zero-fluoroscopy use during ablation could be realized to minimize the long-term risk of EP procedural irradiation in a pediatric population. In 2002, Drago and colleagues used single catheters of EAM to map and ablate right-sided accessory pathways [42]. With a 95% success rate with no complications, zero-fluoroscopy ablation studies gradually emerged to encompass all forms of arrhythmias in various patient populations. A recent literature review of EP ablation procedures analyzed studies over the last 17 years that aimed for near-zero to zero fluoroscopy in 20 trials to treat supraventricular tachycardia (SVT), 10 trials to treat atrial fibrillation (AF), and 4 trials to treat VT [7]. In sum, 93% of 1,989 SVT patients had zero fluoroscopy during their procedure. More specifically, the AF trials included retrospective studies or consecutive enrollment trials to move from near-zero to zero fluoroscopy. From a safety perspective, even though these trials were non-randomized, none showed any significant greater risk of complications. In a new multicenter prospective non-randomized trial, investigators analyzed 1020 SVT patients treated with zero-fluoroscopy guidance against 2040 SVT patients with conservative fluoroscopic imaging [43]. No differences were found comparing procedure times, complication rates, or success rates. Similarly, in an assigned 1:2 ratio consecutive enrollment multicenter trial for VT ablation, 94% of procedures were achieved without fluoroscopy [44]. Five patients out of 163 required fluoroscopy because of needed coronary angiography.

The review by Canpolat and colleagues identified that the most common hindrance preventing the goal of reaching zero-fluoroscopy time was specific physician apprehension to perform the transseptal puncture portion of the procedure [7]. Concomitant ICE-guided transseptal puncture has solidified physician confidence allowing direct real-time visualization of the transseptal needle at the interatrial septum with visualization of left atrial microbubbles as the ablation energy allows safe passage of the transseptal needle into the left atrium. In a multicenter trial of 744 patients undergoing AF ablation, 100% success was achieved transseptal puncture without fluoroscopy, with a 0.5% complication of either pericardial effusion or tamponade [45]. It was unknown if this complication resulted from the puncture or AF ablation. It is interesting to note that concern for lead dislodgement, patients with newly implanted devices lead (< 3 months) were excluded. There was no device interrogation evidence of dislodgement in all 46 patients with CRM devices that underwent AF ablation. Further obstacles to zero fluoroscopy in select patient groups have been identified in those that may require epicardial forms of VT ablation [7, 46].

The learning curve for adopting a fluoroless workflow in electrophysiology procedures has been studied previously. Analyzing historical data, a general learning curve was noted by identifying a decrease in procedure times comparing 1 year to the next while completing fluoroless cryoablation of atrioventricular nodal reentrant tachycardia (AVNRT) [47]. Kochar and colleagues demonstrated that in a single-center experience, a zero-fluoroscopy workflow could be adopted safely for standard radiofrequency ablation procedures, including pulmonary vein isolation (PVI), supraventricular tachycardia, and premature ventricular contractions (PVC) [48]. In their analysis, the steepest learning curve occurred over the first 40 cases of PVI, 20 cases for SVT, and 15 cases for PVCs. A similar single-center, retrospective analysis by Zei and colleagues demonstrated the safety and efficacy of a fluoroscopic reduction workflow for PVI [49]. A significant downward trend in the mean fluoroscopic time was observed, suggesting a rapid learning curve. Experienced operators may likely have learning curves of less than 10 cases [50]. The use of standardized simulation labs or formal training programs should also help accelerate the learning curve and ensure patient safety.

### CRM device implantation procedures are last to phase out fluoroscopy

Studies to employ a workflow of zero-fluoroscopy for implantation of CRM devices lag in technology and the number of studies compared to ablation procedures. Several reasons may account for this shortfall. Unlike the ablation procedures, the implant procedure of CRM devices requires the permanent placement of pacemaker or defibrillator leads within one or more chambers at specific positions. It has already been shown that the two EAM systems are capable of interfacing with pacemaker or defibrillator leads to provide their position within the previously created virtual 3D cardiac image during the implant procedure [30, 51]. The Carto system, however, requires a custom-made connector to provide a workaround to see the device lead tip [51]. ICE probes can already image a lead body, but in most platforms, the lead body is seen in a 2D cross-sectional slice. The CRM leads are constructed differently than EAM catheters, often by different companies, and require separate investigations to achieve FDA approval. To maintain a permanent, stable position, the distal tip of the CRM leads has either passive fixation tines or active fixation deployable screw tips. As will be shown below, and with some modification, both EAM and ICE could also be used directly to replace fluoroscopy in most cases for implantation of CRM devices. In addition, EAM can easily be repurposed to map the great vessels that lead to the heart as well as the coronary sinus (CS) vein and its branches (Figs. 2 and 3).

EAM has been shown to significantly reduce fluoroscopy times with single [52, 53], dual [16, 54, 55], and CRT devices [15, 30, 56]. For traditional CRT devices, the most time-consuming and fluoroscopic-dependent step is the cannulation of the CS os with the guide sheath. This step also necessitates a contrast dye injection with simultaneous cinefluoroscopy to create a fluoroscopic map of the CS vein and branching vessels. Early data from 2012 showed that EAM could be used alongside fluoroscopy to reduce radiation exposure with the implantation of CRT devices [57]. Reduction of fluoroscopy decreased from an average of 16.8 min down to 4.2 min with EAM guidance. However, these implants still needed cinefluoroscopy to map the CS vein and its branches. An Italian multicenter trial expanded upon this technique reducing average fluoroscopy times to 4.1 min with acceptable procedural success and complication rates [15]. Of 125 patients, 122 patients had successful LV lead placements. A total of 5 ventricular lead dislodgements occurred (2 left, 3 right), and one patient was determined to have an asymptomatic CS dissection without pericardial effusion. No significant differences were found for procedure times, the success of LV lead placement or complications compared to historical controls of 250 patients. Procedure times remained the same with or without EAM, ranging from 1.5 to 2.5 h. Huang and colleagues published a workflow utilizing EAM, which resulted in an 86% reduction of ionizing radiation exposure [30]. None of these investigations found any significant change in procedure times or complication rates.

Individual labs have creative, innovative techniques to minimize fluoroscopic use. Despite the excellent reduction of radiation exposure and presumed risk reduction of DSE and DDE, physicians and all lab personnel still need to wear protective lead garments under their sterile gowns for the entirety of the procedure. The high DOE risk to develop an orthopedic disability in the EP personnel remains unchanged. At a probable even higher detrimental risk, the procedural nurses and EP technicians wear lead aprons on average 20–30 min longer than the physician for all cases. They may stand slightly further away from the x-ray equipment, which decreases DDE and DSE compared to the physician. Still, the increased apron-time would be expected to have a significantly greater DOE causing greater MSD. The impact of more prolonged apron-time on the prevalence of MSD is currently unknown for any EP lab personnel. Rolling lead barriers/coats for the physician provide no benefit to associate EP workers.

Very limited case reports and studies have been published that document the complete elimination of fluoroscopy during CRM implant procedures. The first implant utilizing EAM only was performed in 2005 on a patient undergoing AV node ablation with an implant of a single chamber pacemaker with a passive fixation lead [58]. An end-of-case single shot fluoroscopic image confirmed proper positioning. The pacemaker passive fixation lead type and last x-ray shot identified limitations of EAM that cannot identify active fixation screw deployment or lead body slack. This case study was followed by a series of 15 patients implanted with a single-lead VDD (ventricular paced, dual atrial and ventricular sensed, and dual atrial and ventricular response) pacemaker system. Again, passive fixation leads were used with a confirmatory single fluoroscopic shot prior to closure [53]. Compared to historical controls, implantation of single-lead CRM devices with EAM imaging alone did not significantly increase procedure time [16, 53]. However, the procedure time for implantation of dual lead CRM devices (*n* = 3), using only EAM imaging, increased by an average of 21 min. The procedure time in such small studies does not negate the large beneficial prospect that alternate imaging methods eliminate all the three risk categories described above for fluoroscopy. The procedure times would be expected to decrease both from an expected learning curve to the use of alternate imaging tools as well as from some technological improvements that are discussed below. It is even possible that procedure times may achieve shorter durations than fluoroscopy-guided implants.

If EP procedures continue to use fluoroscopy at any step, then the only way to decrease DOE is to severely reduce procedure times. Realistically, to achieve a reduction of DOE for EP physicians and staff, the elimination of fluoroscopy becomes the true objective. Thus, with a focus below to diminish DOE for all EP personnel, all portions of the implant procedure will need safe elimination of fluoroscopy at each step. The steps proposed below are for the complete replacement of fluoroscopy to implant CRM devices.

#### Imaging replacement steps for CRM implants

##### *Vascular access*

**Fig. 4:**
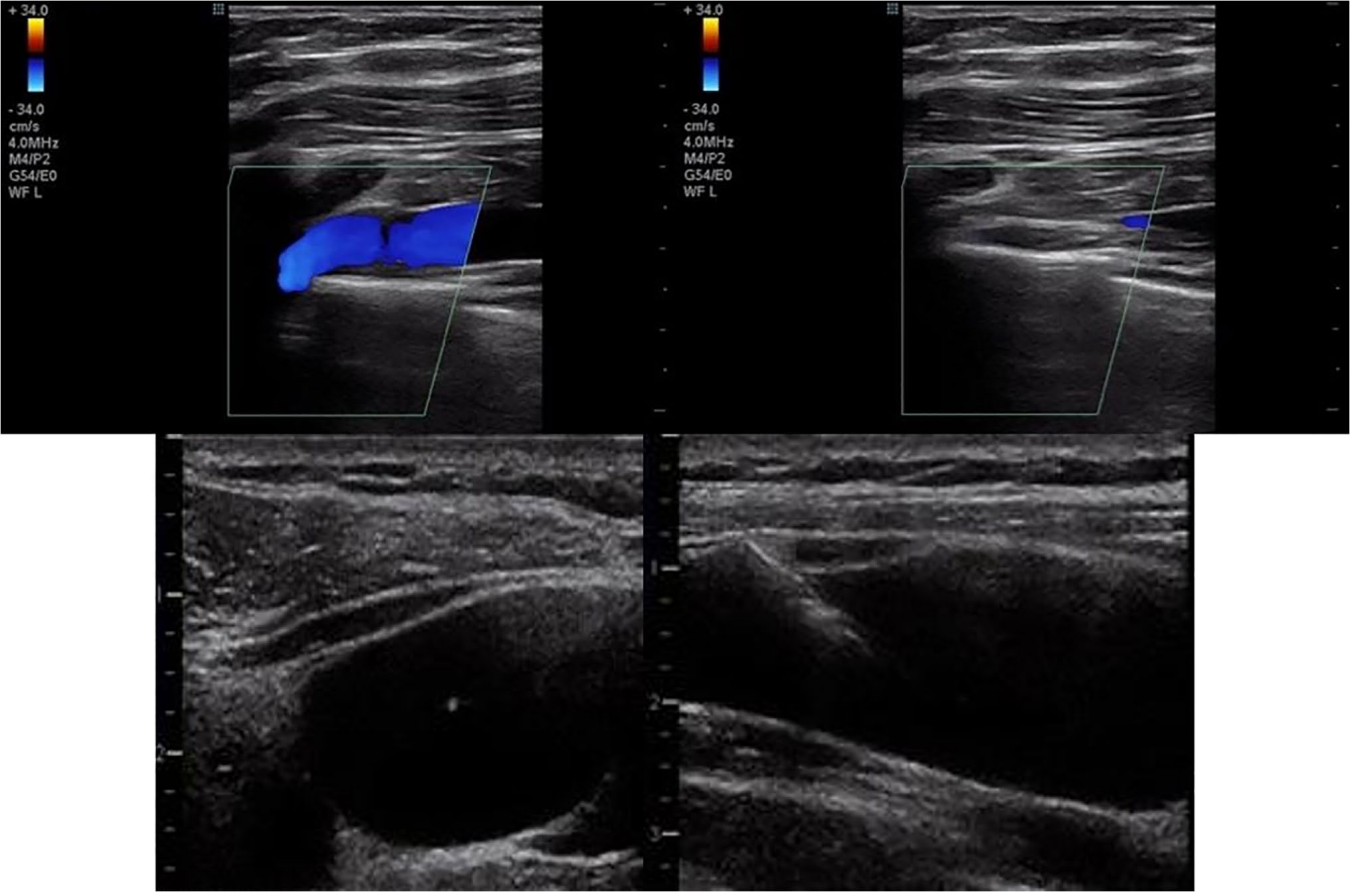
Upper left panel: transcutaneous images with color Doppler flow of left subclavian vein (blue vessel). Upper right panel: the same vessel was confirmed to be venous with ease of compressibility and digital pressure over the lumen. Lower panels (figure obtained with permission from common license) [41]. Transcutaneous ultrasound shows direct visualization of access needle insertion into a central venous vessel to a proper depth

Visualization of the subclavian or axillary can be obtained by transcutaneous ultrasound probe along the deltopectoral groove [59, 60]. Confirmation of blood flow directionality is accomplished with either Doppler color or simply with external pressure. With proper probe alignment, the vascular needle can be observed to enter the vessel lumen without passing through the back wall of the blood vessel or penetrating deeper structures (Fig. 4). Confirmation of the guidewire in an endovascular location can also be visualized with a long-axis image of the target vessel.

##### *3D venous passage to cardiac chambers and virtual structure creation*

Once the first vascular access is obtained, either an EAM mapping catheter or an ICE probe can be advanced to the cardiac chambers to construct anatomy en route to the atrium and ventricle. Alternatively, the imaging access site could be obtained from a different location (femoral vessels). Images obtained would be similar to those of Figs. 2 and 3. Both ICE and EAM can create accurate 3D chamber images. EAM can additionally provide local endocardial surface voltage that may prove to be necessary to implanters. Researchers could utilize this data while creating the chamber structure that might reasonably identify the best permanent landing sites (regions of highest voltage sensed and/or lowest capture threshold) to target as the insertion site for pacing leads. Such data would be relatively fast and easy to obtain. New pacing capture threshold algorithms could be incorporated at common standard landing sites of pacing leads that may identify the most suitable permanent lead position.

##### *Implantation of atrial and ventricular leads and confirmation of slack and helix deployment*

**Fig. 5:**
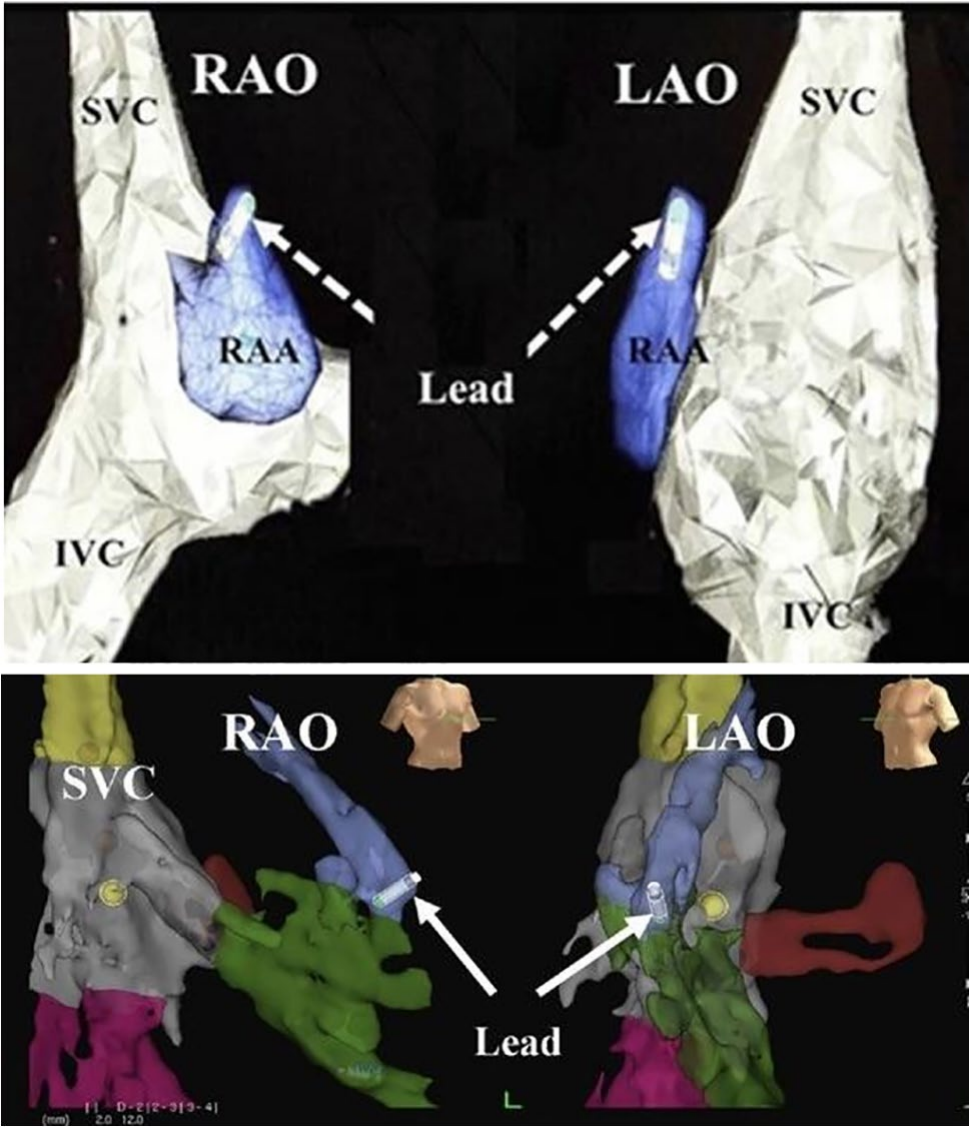
Pacemaker leads are located within the chambers of the heart [16] (reproduced by CC-BY-NC license 5250881328925 from John Wiley and Sons). Top panel, right atrial pacemaker lead in the tip of right atrial appendage (RAA). Bottom panel, right ventricular pacemaker lead in right ventricle at the mid septal position

A review of current literature exposes few reports of single or dual chamber device implantations using zero-fluoroscopy. Guo et al. published a case series of 6 patients describing a zero-fluoroscopy approach to pacemaker implantation [16]. All procedures were performed using the EnSite NavX (St. Jude Medical, MN, USA), which utilizes 3 orthogonal pairs of electrode patches to geometrically create the right atrium and ventricle. The ventricular lead was placed by first obtaining venous access and then introducing the lead into the venous system. An alligator cable was used to connect the lead to the EnSite NavX system, making appropriate adjustments for the lead’s interelectrode distance. Then using the lead much like a mapping catheter, geometry of the superior vena cava, right atrium, and right ventricle was made by moving the pacing lead along the endocardial surface of each respective chamber (Fig. 5). Once the ventricular lead was in a suitable place at the RV apex or RV septum, it was advanced 3–5 cm to account for the required slack. The lead was deployed in the usual fashion. A second set of alligator cables connected the lead to an analyzer to ensure adequate pacing, sensing, and impedance measurements. The leads were then advanced, withdrawn, and rotated to ensure stable measurements. Furthermore, the patient was asked to breathe deeply and cough prior to a final set of measurements.

For patients requiring a dual chamber pacemaker, other techniques were employed to ensure proper right atrial appendage lead placement. First, right femoral venous access was obtained, and a steerable 10 pole catheter was used to make a 3D anatomic model of the right atrium and right atrial appendage. The atrial lead was then advanced from the subclavian vein access site with the lead connected to the EnSite NavX mapping system to visualize the lead tip (Fig. 5). The lead was manipulated into the right atrial appendage and deployed once in a suitable position [16]. The above-mentioned RV lead techniques were performed to ensure proper slack, lead parameters, and lead stability.

Passive or active fixation leads could be imaged similarly to determine real-time position within the cardiac chambers. Since EAM currently has no method of determining lead body slack, ICE imaging might be a preferred imaging tool. However, importance could be placed on developing visible lead bodies just as EAM systems have developed visible sheaths. For example, the VIZIGO® sheath can be seen on the Carto mapping system once it enters the matrix of collected points. Activation screw tip deployment will likely require additional confirmation methods to be developed. In the meantime, a final fluoroscopic image could be utilized to ensure proper slack and helix deployment with staff appropriately shielded behind protective barriers but immediately available to attend to patient needs.

##### *Cannulation of coronary sinus vein with lead delivery sheath*

**Fig. 6:**
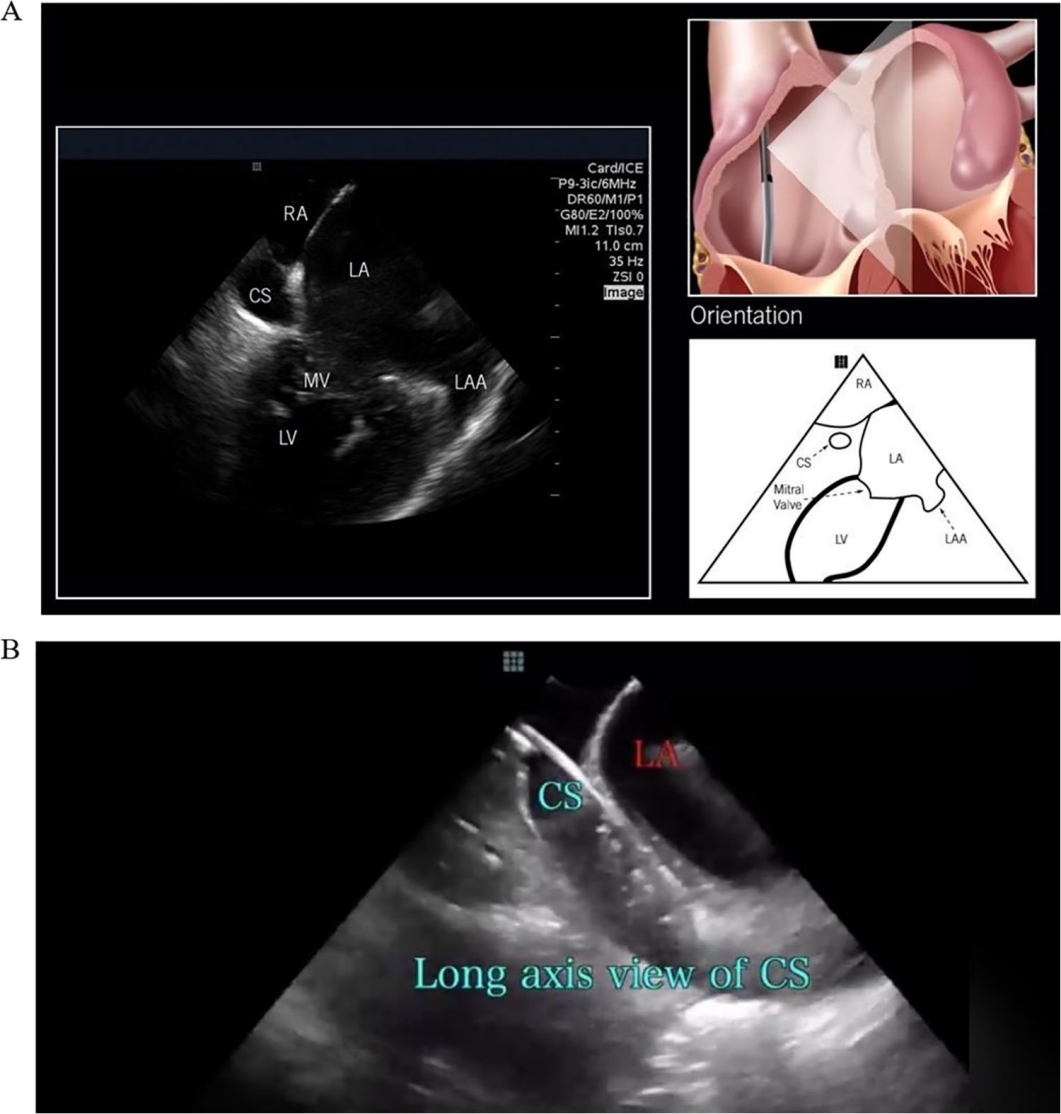
**A** ICE imaging of coronary sinus (CS) and cannulation of the catheter. Left panel, ICE image from within right atrium (RA) shows CS os. Right panel, the orientation of ICE catheter from within RA (from St. Jude brochure of ViewFlex Xtra ICE Catheter). **B** An electrophysiologic wire enters CS in a long-axis view. Image shown is courtesy of Mansour Razminia

Placement of the left ventricular lead is typically the most time-consuming step when implanting a CRT device. As such, fluoroscopic time and radiation exposure can be excessive. Cannulation of the lead delivery sheath into the os of the CS vein using 2D x-ray is based upon positioning a sheath anatomically relative to other fluoroscopically identifiable structures such as the annular “fat stripe.” Then while under continuous fluoroscopy imaging, the posteroseptal atrial septum is probed with soft, flexible wires or catheters. Once the sheath is advanced into the CS vein, it is common practice to inject contrast dye for vein patency confirmation and to define potential target venous branches along the posterolateral left ventricular wall. This step is easily replaced with direct visualization of the CS by ICE imaging and direct insertion of EP catheters (Fig. 6). Once the wire or catheter has been advanced into the CS vein, the lead delivery sheath or sub-selector sheath can be placed over the wire and into the CS vein.

##### *Coronary sinus vein branch identification and lead insertion*

**Fig. 7:**
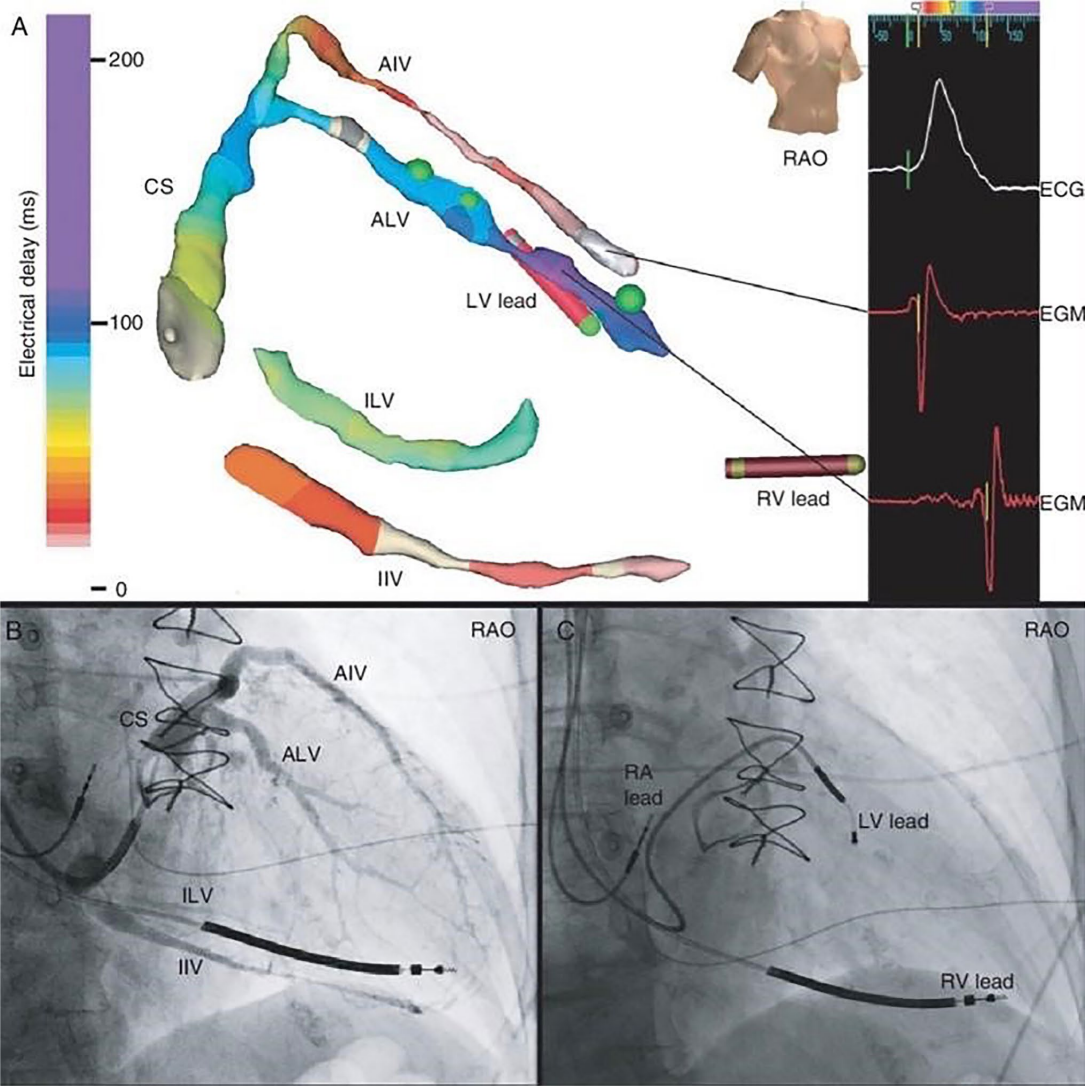
Placement of CS lead via 3D electroanatomic mapping. Activation timing measurements for selecting the best vein branch for resynchronization (reproduced with permission) [61]. IIV, inferior inter-ventricular vein; ILV, inferolateral vein; ALV, anterolateral vein; AIV, anterior inter-ventricular vein

The first study to implant CRT devices without fluoroscopy utilized EnSite NavX EAM in 26 patients [56]. An additional femoral vein access for an EAM mapping catheter allowed passage to the cardiac chambers to construct a 3-dimensional image of the right atrial (RA) appendage and CS. Three sheaths were placed into the left subclavian vein (utilizing ultrasound for access). An active fixation RA lead was placed into the RA appendage. A bipolar pacemaker or defibrillation right ventricular (RV) lead was inserted at the RV apex. An electrophysiologic catheter through a lead delivery sheath was advanced into CS os, mapping along the CS vein and openings of vein branches. This catheter was replaced with the left ventricular (LV) lead and advanced within one of the vein branches. It had been previously shown that a soft VisionWire (Biotronik) used as a guidewire protruding through the lead lumen could safely map CS vein branches anatomically and test the underlying substrate for acceptable pacing sites (Fig. 7) [61]. The distal tip of the LV wire is virtually imaged by an alligator clip connection from the lead pin to the EAM system. Successful deployment in 24 of 26 LV leads was achieved [56]. The left subclavian vein was obstructed in one patient. A successful device implant from the right subclavian access was also noted with this method. In the second patient, fluoroscopy identified a Thebesian valve obstruction. Once a guidewire was passed beyond the obstruction, the LV lead was placed without further fluoroscopy. Note that with this method of implant, proper lead body slack is unable to be determined.

### Qualitative imaging comparison

Table 1 provides a comparison between imaging modalities to help identify step-specific deficiencies that industry partners might advance to make zero-fluoroscopy implants of all new devices a reality.

**Table 1:** Comparison of imaging modalities for implant of CRT device. *Echo TC*, transcutaneous; *ICE*, intracardiac. − , poor or no visualization; + , visualized adequately; − **/ + **, some case reports identified capability; + ** + **, best visualization

Visualization	Fluoroscopy	Electroanatomic mapping (EAM)	Echo TC/ICE	EAM + echo
Dimension	2D	3D	2D/3D/4D	3D
Vascular access	** + **	** − **	** + + **	** + + **
Lead tip position chamber	** − **(limited)	** + + **	** + **	** + + **
Active fixation screw	** + **	** − **	** − **	** − **
CS os	** − **	** + **	** + **	** + + **
CS vein cannulation	** − **	** + **	** + **	** + + **
CS vein branches	Only with contrast	** + **	** − **	** + **
LV lead deployment	** + **	** + **	** − **	** + **
Lead slack	** + **	** − **	** + **	** + **
Old leads	** + **	** − **	** + **	** + **
Effusion/tamponade	** − / + **	** − **	** + **	** + **
Pneumothorax	** + **	** − **	** − / + **	** − / + **

Each modality of imaging has different advantages and disadvantages. However, combination ICE plus EAM provides superior imaging overall benefit that could completely eliminate the need for fluoroscopy for initial implantation. Final positions of leads could be confirmed with a post-procedure x-ray providing future comparisons in case of concern for dislodgement or other complications.

## Needed technological improvements

Several deficiencies could easily be overcome with existing technology. Most obvious is the need to image the lead body and active lead screw deployment. At least two main avenues of approach will help achieve the fluoroless goal. Innovation of the leads and/or the imaging equipment itself may be required. Placing sensors along the distal 5–10 cm of the pacing lead body will allow adequate 3D virtual imaging, providing the operator knowledge of sufficient lead slack and proper active fixation screw protrusion. During the EAM of cardiac chambers, voltage and rapid pacing algorithms could predetermine the best landing sites for permanent pacing, saving time, and possible complications. New electrical testing algorithms could be devised to confirm proper screw deployment. New coronary sinus vein branch mapping tools might include small gauge sensor-laden flexible and deflectable fiber that allow adequate mapping, lead guidance, and capture threshold testing. ICE probes could be designed to allow 360° rotation as opposed to deflection which could otherwise result in dislodgement of a lead just implanted. During the probe rotation, a virtual image of the lead could be accurately placed in the virtual 3D chamber. A 4D ICE probe would allow real-time imaging of all leads within the cardiac chambers.

To our knowledge, there is no current research and development in placing sensors on pacing or defibrillator leads to allow for EAM and delivery without the use of fluoroscopy. We do, however, know from the MediGuide™ technology (St. Jude Medical Inc., St. Paul, MN) that sensors mounted on intracardiac devices (electrophysiology catheters) can be visualized in real time, thus minimizing fluoroscopy exposure [62–64]. If this concept is applied in the development of sensors placed at the tip of pacing and defibrillator leads, we could potentially implant devices without fluoroscopy. More research would be needed to understand how the leads would handle and perform with the addition of mapping sensors placed at their tip. Most certainly, any new technology placed within implanted leads will require clinical testing for FDA approval. Time costs and financial costs will present as barriers. Even well-intentioned decisions to save a mechanically balanced method from a previous FDA-approved lead design could backfire. The defibrillator lead recall of 2011 involved a silicone insulation breach with externalization of the conductor cables that could cause electrical failure (https://www.accessdata.fda.gov/scripts/cdrh/cfdocs/cfres/res.cfm?id=105847). In 10 of 20 new Riata models, inert or electrically inactive filler cables remained in the lead body design to maintain mechanical stability. Unfortunately, the stabilizing filler cable could break its distal attachment and extrude from the insulation breach as well [65]. An extruded filler cable extrusion escaped detection by standard device surveillance and added to the confusion of how best to manage a costly recall.

Concerning the recently developed leadless pacing devices, fluoroscopy is still a specific requirement for placement. In the case of the currently available Medtronic device, fluoroscopy is used to ensure proper placement by confirming the attachment of the device tines. Fluoroscopy is also used to ensure proper deployment of the yet-to-be commercially released devices from Abbott and Boston Scientific. Using intracardiac ultrasound as an alternative imaging strategy for placement is not currently an option. However, these device companies can and should develop technology within the leadless implant that would allow for detection either under ultrasound or through electromagnetic or impedance field 3D mapping. Such technology could be deployed to leadless devices along with other possible engineering designs that will allow fluoroless imaging of these devices. It may best to advise the FDA, industry, and academic medical institutions to veer their research from the very start toward a direction that needs to be accomplished without imaging by irradiation.

## Imaging costs

A genuine concern about utilizing fluoroscopy-less technologies such as ICE and 3D mapping is the cost. Cost is a true upfront limitation to widespread adoption of fluoroscopy-free device implantation. Current financial reimbursement to physicians and hospitals is being decreased, forcing decisions that favor cost-cutting, even if at the expense of longer case times, worse efficiency, and overall patient and provider safety. Although different hospital systems can negotiate a range of prices for these technologies, new ICE catheters are approximately $1000 with some discount if using reprocessed equipment. 3D mapping patches also cost roughly $500–1000 per patient. Navigation enabled catheters cost approximately $1000, creating an overall cost per case of $2,500–$3000. In traditional lab systems, these per-case costs are in addition to the fluoroscopy systems that are in place. Single and biplane fluoroscopy systems cost upward of 1.5–2 million dollars and $5,000–10,000 monthly for routine upkeep and preventive maintenance. However, if both ablation and implant of CRM devices could be accomplished in future EP labs that no longer require an upfront fluoroscopy system cost, then the individual case costs would be offset.

In addition, interest is exponentially increasing in the cost analysis benefits of both minimal fluoroscopy and zero-fluoroscopy techniques. Unfortunately, in the setting of device implants, some of the detrimental consequences are a zero-sum game. We have previously described the DSE, DDE, and DOE that ionizing radiation and lead aprons can accumulate over the years in the EP lab. Even if decreasing fluoroscopy to a few minutes per case, the physician and staff are required to wear lead aprons for the entirety of the case without any mitigation of the DOE risks. In addition, the authors of the multicenter, randomized controlled NO-PARTY trial, who compared fluoroscopy-guided or minimal fluoroscopy-guided EP Study in SVT ablation with the EnSite NavX system, concluded that the additional cost of incorporating near-zero fluoroscopy was offset by the reduction in cancer afforded by this technique [66]. We should not throw the baby out with the bathwater. The pursuit toward zero-fluoroscopy device implantation should not be discounted due to the current cost of lab setups and current technology. If fluoroscopy had not been the first imaging method applied to EP procedures, and EAM and ICE were the initial imaging tools, then fluoroscopy may have never progressed beyond a very limited C-arm use. In fact, with continued improvements in image and catheter refinement and early adoption in academic training programs, the requirement for costly fluoroscopic imaging in future EP labs may not be required and certainly not the gold standard.

## Conclusion

The exceedingly high prevalence of orthopedic disability among EP physicians and their lab personnel should be greatly feared. There is an additional concern beyond the scope of retirement in pain. DOE is a rapidly growing deterrence to attract the brightest physicians to, arguably, the most biophysically complex healthcare field. The era to usher in fluoroless EP labs is at hand. The two categories of EP procedures that had relied upon fluoroscopic imaging include ablation of arrhythmias and implant of CRM devices. A review has been presented of the technology and multiple studies that have proven the safety, effectiveness, efficiency, and short learning curves that can eliminate fluoroscopy use in ablation procedures. Much of the same technology can be easily pivoted to evolve implant procedures of CRM devices. Shortcomings of fluoroless ablation procedures would include epicardial ablation or alcohol injection of the vein of Marshall, where fluoroscopy use may still be required but in a far more limited role. Rescue fluoroscopy, at least by C-arm, should remain available for EP labs to assess for potential complications.

We have identified methods to eliminate fluoroscopy use at each specific step of the implant procedure of CRM devices. Most of these methods already use FDA-approved equipment that requires additional clinical studies to provide further safety measures, time-saving steps, and shorter learning curves. We call for new studies, new apron-time metrics, new simulation labs, new learning curve assessments, and new priorities in fellowship training programs. We have identified that the DOE risks are of such a high magnitude that this categorization of risk needs to be placed alongside and maybe in front of the DDE and DSE risks of fluoroscopy. Cost barriers for new lead design, in addition to risks of recalls, perhaps too high to overcome, thus might divert innovation toward new imaging refinements and modalities that can track lead advancement, slack, and helix deployment. The “cure” that lead aprons can prevent the development of cancer for 1 in 92 physicians is overtaken by the fact that 1 in 3 EP physicians will develop temporary or permanent orthopedic disability. Fluoroscopy is an obsolete, harmful tool that creates a 2D gray shadow world with zero electrophysiologic information, when what is required is a 3D colored electrophysiologic world. It is quite feasible that the first company to provide efficient, safe, and accurate visualizing methods to implant CRM devices will have a significant market advantage.

In a stooped posture, EP physicians now begging for methods and training to eliminate fluoroscopy is not an unexpected paradigm shift. Everything old is new again.

*Aegrescit medendo* is replaced appropriately by *cur ate ipsum*.

Physician heals thyself!

## Supplementary Information

Below is the link to the electronic supplementary material.

Supplementary file1 (MP4 21377 KB)
