# Correction to: Aegrescit medendo: orthopedic disability in electrophysiology—call for fluoroscopy elimination—review and commentary

**DOI:** 10.1007/s10840-022-01248-3

**Published:** 2022-05-26

**Authors:** Donald S. Rubenstein, Benjamin B. Holmes, Joseph A. Manfredi, Matthew S. McKillop, Peter C. Netzler, Chad C. Ward

**Affiliations:** 1 Carolina Cardiology Consultants–EP Division, Prisma Health, 701 Grove Rd., Greenville, SC 29605 USA; 2 Baptist Medical Center Jacksonville, 800 Prudential Dr., Jacksonville, FL 32207 USA

**Correction to: Journal of Interventional Cardiac Electrophysiology**

**https://doi.org/10.1007/s10840-022-01173-5**

Correction to original version of Figure 6B caption which now attributes source image provided in courtesy by Dr. Mansour Razminia. The authors regret this error.

Figure 6 caption should have appeared as shown below.

**A** ICE imaging of coronary sinus (CS) and cannulation of the catheter. Left panel, ICE image from within right atrium (RA) shows CS os. Right panel, the orientation of ICE catheter from within RA (from St. Jude brochure of ViewFlex Xtra ICE Catheter). **B** An electrophysiologic wire enters CS in a long-axis view. Image shown is courtesy of Mansour Razminia.

The original article has been corrected.

